# Predictive model for left main coronary artery or triple vessel disease in patients with chronic coronary syndromes

**DOI:** 10.2478/abm-2024-0024

**Published:** 2024-09-20

**Authors:** Piyanop Nuchanat, Komsing Methavigul

**Affiliations:** Department of Cardiology, Central Chest Institute of Thailand, Nonthaburi 11000, Thailand

**Keywords:** chronic coronary syndromes, high risk, left main, prediction, three-vessel disease

## Abstract

**Background:**

Data about prediction of left main coronary artery disease (LMCAD)/three-vessel disease (TVD) in patients with chronic coronary syndromes (CCS) are lacking.

**Objectives:**

This study aimed to develop a model for predicting patients at risk of LMCAD/TVD.

**Methods:**

This study used retrospective data from patients with CCS scheduled for invasive coronary angiography (ICA) and who were retrospectively recruited between January 2018 and December 2020. Predictors were obtained and analyzed by using logistic regression analysis, and generated the prediction score. The sensitivity, specificity, positive predictive value (PPV), and negative predictive value (NPV) were calculated. The cut-off value and area under the curve (AUC) were analyzed by using the receiver operating characteristic (ROC) curve.

**Results:**

We recruited 162 patients with CCS. There were 75 patients in the non-LMCAD/TVD and 87 patients in the LMCAD/TVD groups. After the multivariate analysis, new onset of heart failure (HF) or left ventricular systolic dysfunction (LVSD) and suspected CAD, ST elevation (STE) in aVR, STE in V_1_ and lateral ST depression (STD) were associated with increased risk of LMCAD/TVD. Based on these 4 predictors, the prediction score was created. The cut-off value of the prediction score by using ROC curve analysis was 3.0. The sensitivity, specificity, PPV, and NPV were 71.26%, 86.67%, 86.11%, and 72.22%, respectively, with an AUC of 0.855.

**Conclusions:**

The CCS patients with new onset of HF or LVSD and suspected CAD, STE in aVR, and STE in V_1_ and lateral STD were associated with increased risk of LMCAD/TVD. The novel prediction score could predict LMCAD/TVD in those patients with acceptable sensitivity, specificity, PPV, and NPV.

Coronary artery disease (CAD) is categorized as acute coronary syndromes (ACS) and chronic coronary syndromes (CCS) [1,2,3]. Recent European guidelines recommend invasive coronary angiography (ICA) for the diagnosis of patients with high clinical likelihood, severe symptoms after antianginal therapy, or high risk of cardiovascular (CV) events [3].

High-risk patients with CCS are defined as those having three-vessel disease (TVD) with proximal stenoses, left main CAD (LMCAD), or proximal left anterior descending artery [3]. About 4%–6% of the patients with LMCAD undergo ICA [4]. In addition, approximately 70% of those patients also have multiple coronary artery stenosis, leading to a large ischemic area [5, 6].

Previous studies have shown that patients with ACS having LMCAD and TVD were associated with electrocardiographic changes such as ST-segment elevation (STE) in aVR lead ≥0.05 mV [7, 8]; number, extent, or location of leads having ST-segment depression (STD) [9, 10], and heart failure (HF) at clinical presentation [11]. Age, male sex, diabetes, blood pressure, hyperlipidemia, obesity, smoking history, heart rate, history of ACS or ICA, and peripheral artery disease have also been studied and associated with LMCAD and TVD [10, 11]. However, there is a lack of data in patients with CCS. This study aimed to develop a predictive model for predicting patients with CCS at risk of LMCAD and TVD.

## Methods

This study used the retrospective data from patients with clinically suspicious CCS, aged 18 years or more, and scheduled for ICA. Patients were recruited at the Central Chest Institute of Thailand between January 2018 and December 2020. The exclusion criteria included patients with bundle branch block including right bundle branch block (RBBB), left bundle branch block (LBBB) or intraventricular conduction disturbances (IVCD), ventricular paced rhythm, moderate-to-severe valvular heart disease, stress cardiomyopathy, myocarditis, hypertrophic cardiomyopathy, hypertensive emergency, severe anemia (hemoglobin concentration <8 g/dL), myeloproliferative disorders, pericarditis, Brugada syndrome, or electrolyte abnormalities (hypokalemia, hyperkalemia, hypomagnesemia, hypocalcemia).

The baseline demographic data, 12-lead electrocardiography (ECG), and angiographic data were collected from the medical records. Predictors such as age, estimated glomerular filtration rate (eGFR), new onset of HF or left ventricular systolic dysfunction (LVSD) and suspected CAD, number of leads having STD, summation of leads having STD, STE in aVR, STE in V_1_, anterior STD, lateral STD, and inferior STD were obtained and analyzed.

STD and STE were defined as STD and STE ≥0.05 mV, respectively. Anterior STD was defined as STD ≥2 leads in lead V_1_–V_4_. Lateral STD was defined as STD ≥2 leads in lead I, aVL, V_5_–V_6_. Inferior STD was defined as STD ≥2 leads in lead II, III, aVF. The extent of STD or STE was measured from TP segments at 80 ms after J point in the resting 12-lead ECG.

The angiograms were classified as LMCAD (≥50% stenosis) or TVD (≥70% stenosis), and non-LMCAD/TVD disease.

This study complied with the Declaration of Helsinki, the International Conference on Harmonization for Good Clinical Practice Guidelines, and designed following the recommendations of the STROBE and TRIPOD statements [12, 13]. The study protocol was approved by the Human Research Ethics Committee of the Central Chest Institute of Thailand (certificate of approval no. 018/2564).

### Statistical analysis

We specified 0.05 for type I error and 0.20 for type II error; hence, the power of this study was 0.80. The estimated LMCAD/TVD was 39% in patients with STE in aVR and 18% in patients without STE in aVR, so the odds ratio (OR) was 2.91 [10]. The ratio between the two groups was 1. The sample distribution was binomial. The sample size was calculated by using G*power version 3.1.9.7 using the methods of Faul et al. [14] and Demidenko [15, 16]. The sample size in this study was 81 patients and included the missing data in each group; so, the total number of patients was 162.

We analyzed the baseline demographic data with descriptive statistics. Categorical data are presented as frequency and percentage and compared by using the chi-squared test or Fisher exact test. Continuous data are presented as mean ± standard deviation (SD) and compared by using independent *t*-test.

All variable values were used for the univariate and multivariate analyses, and the logistic regression analysis was employed to determine the predictors associated with LMCAD/TVD, to obtain the OR and 95% confidence intervals (CIs), and to generate the prediction score. The sensitivity, specificity, positive predictive value (PPV), and negative predictive value (NPV) were calculated. The cut-off value and area under the curve (AUC) were analyzed by using the receiver operating characteristic (ROC) curve. *P* < 0.05 was considered as the statistical significance.

## Results

We recruited 162 patients with clinically suspicious CCS eligible by using sample size calculation with simple random sampling. The average age was 66.1 ± 11.5 years. About half of those patients were males. There were 75 patients in the non-LMCAD/TVD and 87 patients in the LMCAD/TVD groups. About half of those patients had diabetes mellitus and most patients had hypertension and dyslipidemia. The average left ventricular ejection fraction (LVEF) was 56.1 ± 16.3%. About one-third of those patients had STE in aVR or V_1_. The baseline characteristics are shown in **Table 1**.

**Table 1.** Baseline characteristics of the patients

**Demographic data**	**All patients (n = 162)**	**Non-LMCAD/TVD (n = 75)**	**LMCAD/TVD (n = 87)**	***P***
Age (years), Mean ± SD	66.1 ± 11.5	63.7 ± 10.8	68.1 ± 11.8	0.02*
Male sex, n (%)	91 (56.2)	43 (57.3)	48 (55.2)	0.78
eGFR, Mean ± SD	72.88 ± 21.98	76.68 ± 22.17	69.60 ± 21.40	0.04*
LVEF (%), Mean ± SD	56.1 ± 16.3	59.8 ± 14.3	53.0 ± 17.3	<0.01*
New onset of HF or LVSD and suspected CAD	59 (36.4)	15 (20.0)	44 (50.6)	<0.01*
Medical history, n (%)				
Diabetes mellitus	77 (47.5)	31 (41.3)	46 (52.9)	0.14
Hypertension	135 (83.3)	60 (80.0)	75 (86.2)	0.29
Dyslipidemia	135 (83.3)	61 (81.3)	74 (85.1)	0.53
Obesity	79 (48.8)	41 (54.7)	38 (43.7)	0.16
History of HF	47 (29.0)	13 (17.3)	34 (39.1)	<0.01*
Previous MI	108 (66.7)	42 (56.0)	66 (75.9)	<0.01*
Prior PCI	64 (39.5)	29 (38.7)	35 (40.2)	0.84
Prior CABG	9 (5.6)	1 (1.3)	8 (9.2)	0.04*
Peripheral artery disease	2 (1.2)	0 (0.0)	2 (2.3)	0.50
Previous stroke/TIA	5 (3.1)	3 (4.0)	2 (2.3)	0.66
Chronic kidney disease	44 (27.2)	17 (22.7)	27 (31.0)	0.23
Current medication, n (%)				
Aspirin	155 (95.7)	69 (92.0)	86 (98.9)	0.05
P2Y_12_ inhibitors	120 (74.1)	51 (68.0)	69 (79.3)	0.10
Beta-blocker	130 (80.2)	58 (77.3)	72 (82.8)	0.39
ACEI/ARB	105 (64.8)	51 (68)	54 (62.1)	0.43
Non-dihydropyridine CCB	1 (0.6)	0 (0.0)	1 (1.1)	0.35
Nitrates	81 (50.0)	29 (38.7)	52 (59.8)	<0.01*
Statin	156 (96.3)	72 (96.0)	84 (96.6)	1.00
Mineralocorticoid antagonist	18 (11.1)	3 (4.0)	15 (17.2)	<0.01*
Resting ECG, n (%)				
Number of leads having STD, Mean ± SD	2.48 ± 2.66	1.09 ± 1.80	3.68 ± 2.70	<0.01*
Summation of leads having STD (mV), Mean ± SD	0.37 ± 0.56	0.13 ± 0.24	0.58 ± 0.66	<0.01*
STE in aVR, n (%)	51 (31.5)	6 (8.0)	45 (51.7)	<0.01*
STE in V_1_, n (%)	60 (37.0)	13 (17.3)	47 (54.0)	<0.01*
Anterior STD, n (%)	14 (8.6)	3 (4.0)	11 (12.6)	0.06
Lateral STD, n (%)	67 (41.1)	9 (12.0)	58 (66.7)	<0.01*
Inferior STD, n (%)	25 (15.4)	6 (8.0)	19 (21.8)	<0.01*

ACEI, angiotensin-converting enzyme inhibitors; ARB, angiotensin receptor antagonist; CABG, coronary artery bypass graft; CAD, coronary artery disease; CCB, calcium channel blocker; ECG, electrocardiography; eGFR, estimated glomerular filtration rate; HF, heart failure; LMCAD, left main coronary artery disease; LVEF, left ventricular ejection fraction; LVSD, left ventricular systolic dysfunction; MI, myocardial infarction; PCI, percutaneous coronary intervention; SD, standard deviation; STD, ST-segment depression; STE, ST-segment elevation; TIA, transient ischemic attack; TVD, three-vessel disease.

* *P* < 0.05 was considered as the statistical significance.

Predictors were analyzed by using the univariate analysis and illustrated that age, new onset of HF or LVSD and suspected CAD, number of leads having STD, summation of leads having STD, STE in aVR, STE in V_1_, anterior STD, lateral STD, and inferior STD significantly increased the risk of LMCAD/TVD, while eGFR significantly decreased the risk of LMCAD/TVD (*P* < 0.05). After the multivariate analysis, 4 predictors were associated with an increased risk of LMCAD/TVD. Those predictors included new onset of HF or LVSD and suspected CAD, STE in aVR, STE in V_1_, and lateral STD (*P* < 0.05) as shown in **Table 2**. Based on these 4 predictors, the prediction score was created to predict the risk of LMCAD/TVD in **Table 3**.

**Table 2.** Multivariate logistic regression of the study population

**Predictors**	**Univariate analyses**		**Multivariate analyses**	
	
	**OR (95% CI)**	***P***	**OR (95% CI)**	***P***
Age (years)	1.035 (1.01–1.06)	0.02*	1.03 (0.99–1.08)	0.16
eGFR	0.99 (0.97–0.999)	0.04*	1.02 (1.00–1.05)	0.09
New onset of HF or LVSD and suspected CAD	4.09 (2.02–8.28)	<0.01*	3.04 (1.23–7.50)	0.02*
Number of leads having STD	1.59 (1.35–1.87)	<0.01*	0.77 (0.41–1.42)	0.40
Summation of leads having STD	28.74 (8.26–99.97)	<0.01*	1.99 (0.05–73.80)	0.71
STE in aVR	12.32 (4.84–31.36)	<0.01*	5.56 (1.40–22.16)	0.02*
STE in V_1_	5.60 (2.70–11.65)	<0.01*	3.48 (1.37–8.88)	0.01*
Anterior STD	3.47 (0.93–12.96)	0.06	2.32 (0.30–18.28)	0.42
Lateral STD	14.67 (6.42–33.53)	<0.01*	8.41 (1.51–46.88)	0.02*
Inferior STD	3.21 (1.21–8.54)	0.02*	1.05 (0.20–5.63)	0.96

95% CI, 95% confidence interval; CAD, coronary artery disease; eGFR, estimated glomerular filtration rate; HF, heart failure; LVSD, left ventricular systolic dysfunction; OR, odds ratio; STD, ST-segment depression; STE, ST-segment elevation.

* *P* < 0.05 was considered as the statistical significance.

**Table 3.** The prediction scores

**Predictors**	**Point**
New onset of HF or LVSD and suspected CAD	1
STE in V_1_	1
STE in aVR	2
Lateral STD	3

CAD, coronary artery disease; HF, heart failure; LVSD, left ventricular systolic dysfunction; STD, ST-segment depression; STE, ST-segment elevation.

**Figure 1.**
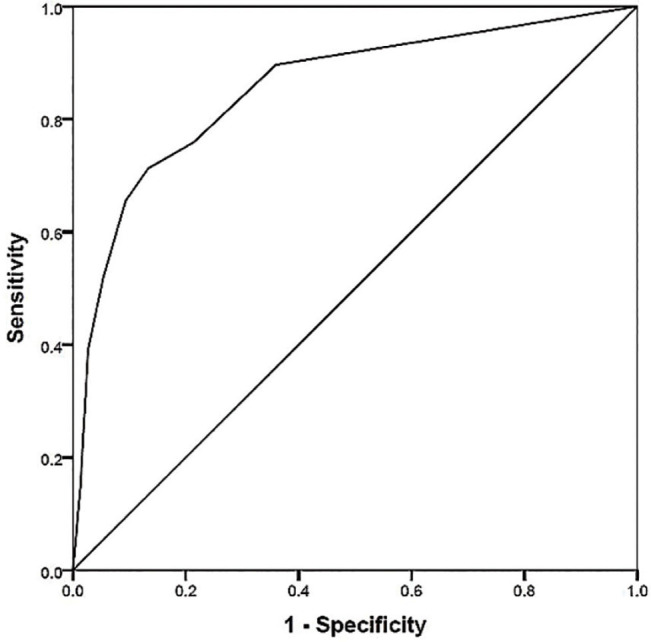
ROC curve of the prediction score. ROC, receiver operating characteristic.

The cut-off value of the prediction score by using ROC curve analysis was 3.0. The sensitivity, specificity, PPV, and NPV were 71.26% (95% CI: 60.57%–80.46%), 86.67% (95% CI: 76.84%–93.42%), 86.11% (95% CI: 75.94%–93.13%), 72.22% (95% CI: 61.78%–81.15%), respectively, with an AUC of 0.855 (95% CI: 0.796–0.914). The ROC curve is shown in **Figure 1**.

## Discussion

To date, previous studies have shown that ACS patients with LMCAD and TVD have been associated with several ECG changes such as STE in aVR, number and location of leads, extent of STD, and CV risk factors. However, there have been no data in patients with CCS undergoing ICA. This study showed that these patients with LMCAD and TVD undergoing ICA were significantly related to new onset of HF or LVSD and suspected CAD, STE in V_1_ or aVR, and lateral STD.

A previous study showed that HF was the predictor of LMCAD/TVD in patients with ACS (OR: 32.50) [11]. In addition, this study showed that there was a statistically significant association between HF and LMCAD/TVD in patients with CCS (OR: 3.04) as well. Although HF in this study included LVSD from suspected CAD, it had less association with LMCAD/TVD than HF in ACS trials. Based on current knowledge, patients with ACS have higher risk compared with those patients with CCS. This may be an explanation for the lower association with LMCAD/TVD in this study. Interestingly, STE in V_1_ during stress test was a predictor of LMCAD/TVD (OR: 4.3) [11], while this study showed that STE in V_1_ during resting ECG was associated with LMCAD/TVD in patients with CCS (OR: 3.21). Symptomatic patients with STE in V_1_ during resting ECG may be high-risk patients in ACS settings as well as CCS patients.

Moreover, a previous study showed that ST-deviations in aVR can predict severity, infarction volume, and prognosis in patients with acute myocardial infarction (MI) [17]. In addition, the meta-analysis has demonstrated that patients with non-ST-elevation ACS and STE in aVR were significantly associated with LMCAD, and a higher extent of STE was a higher probability of LMCAD [8]. This study showed that STE in aVR was associated with LMCAD/TVD with statistical significance in CCS patients; so, STE in aVR can be used for predicting LMCAD in both patients with ACS and CCS.

A previous study in patients with ACS showed that anterior STD, lateral STD, and inferior STD were related to the likelihood of having LMCAD/TVD [10]. This study showed that only lateral STD was associated with LMCAD/TVD with statistical significance, while anterior STD and inferior STD increased the probability of having LMCAD/TVD without statistical significance. The wide 95% CI of patients with anterior or inferior STD due to the small number of patients considered may be the explanation for the non-statistically significant predictors of LMCAD/TVD in this study. Larger studies are required for identifying these patients in future.

The prediction score including new onset HF or LVSD and suspected CAD, STE in V_1_, STE in aVR, and lateral STD was developed. The ROC curve showed a cut-off value of 3.0 with a specificity and sensitivity of 86.67% and 71.26%, respectively. The AUC was 0.855. This study was the first study showing the prediction score used in patients with CCS undergoing ICA. This score can be used to select patients with high CV risk for ICA in future.

However, this study has several limitations. First, patients in this trial were studied in a tertiary hospital, and all patients were scheduled for ICA, indicating a higher CV risk and more severe CAD. This outcome might not be applied in the lower CV risk patients with CCS. Second, this is a retrospective chart review of patients having suspected CCS. Hence, some ACS patients might have been confused with more severe CCS patients. However, the findings in the coronary angiograms can be used for differentiating the CCS from the ACS settings. Finally, this study had a small patient number and selection bias cannot be excluded. Nevertheless, the prediction score can predict the probability of LMCAD/TVD in CCS patients with acceptable sensitivity and specificity.

## Conclusion

The CCS patients with new onset of HF or LVSD and suspected CAD, STE in aVR, STE in V_1_ and lateral STD were associated with an increased risk of LMCAD/TVD. The novel prediction score described here can predict LMCAD/TVD in those patients with acceptable sensitivity, specificity, PPV, and NPV.
